# Imaging and characterization of optical emission from *ex vivo* tissue during conventional and UHDR PBS proton therapy

**DOI:** 10.1088/1361-6560/ad2ee6

**Published:** 2024-03-18

**Authors:** Roman Vasyltsiv, Mahbubur Rahman, Joseph Harms, Megan Clark, David J Gladstone, Brian W Pogue, Rongxiao Zhang, Petr Bruza

**Affiliations:** 1 Thayer School of Engineering, Dartmouth College, Hanover, NH, United States of America; 2 UT Southwestern Medical Center, Dallas, TX, United States of America; 3 Department of Radiation Oncology, University of Alabama at Birmingham, Birmingham, AL, United States of America; 4 Department of Medical Physics, University of Wisconsin-Madison, Madison, WI, United States of America; 5 Department of Radiation Oncology, New York Medical College, Valhalla, NY, United States of America

**Keywords:** cherenkov, proton therapy, optical imaging, UHDR FLASH, luminescence

## Abstract

*Objective*. Imaging of optical photons emitted from tissue during radiotherapy is a promising technique for real-time visualization of treatment delivery, offering applications in dose verification, treatment monitoring, and retrospective treatment plan comparison. This research aims to explore the feasibility of intensified imaging of tissue luminescence during proton therapy (PT), under both conventional and ultra-high dose rate (UHDR) conditions. *Approach*. Conventional and UHDR pencil beam scanning (PBS) PT irradiation of fresh *ex vivo* porcine tissue and tissue-mimicking plastic phantom was imaged using intensified complementary metal-oxide-semiconductor(CMOS) cameras. The optical emission from tissue was characterized during conventional irradiation using both blue and red-sensitive intensifiers to ensure adequate spectral coverage. Spectral characterization was performed using bandpass filters between the lens and sensor. Imaging of conventional proton fields (240 MeV, 10 nA) was performed at 100 Hz frame rate, while UHDR PBS proton delivery (250 MeV, 99 nA) was recorded at 1 kHz frame rate. Dependence of optical emission yield on proton energy was studied using an optical tissue-mimicking plastic phantom and a range shifter. Finally, we demonstrated fast beam tracking capability of fast camera towards *in vivo* monitoring of FLASH PT. *Main results*. Under conventional treatment dose rates optical emission was imaged with single spot resolution. Spot profiles were found to agree with the treatment planning system calculation within >90% for all spectral bands and spot intensity was found to vary with spectral filtration. The resultant polychromatic emission presented a maximum intensity at 650 nm and decreasing signal at lower wavelengths, which is consistent with expected attenuation patterns of high fat and muscle tissue. For UHDR beam imaging, optical yield increased with higher proton energy. Imaging at 1 kHz allowed continuous monitoring of delivery during porcine tissue irradiation, with clear identification of individual dwell positions. The number of dwell positions matched the treatment plan in total and per row showing adequate temporal capability of iCMOS imaging. *Significance*. For the first time, this study characterizes optical emission from tissue during PT and demonstrates our capability of fast optical tracking of pencil proton beam on the tissue anatomy in both conventional and UHDR setting. Similar to the Cherenkov imaging in radiotherapy, this imaging modality could enable a seamless, independent validation of PT treatments.

## 1. Introduction

Surface luminescence imaging allows for direct real-time optical visualization of treatment delivery during external beam radiotherapy (EBRT) and has been primarily applied in the form of Cherenkov imaging (Jarvis *et al* 2014). As a significant portion of the radiotherapeutic energy spectrum lies above the minimum energy threshold for Cherenkov emission (Glaser *et al* 2014), both primary (in case of electron RT) and secondary (electron and photon RT) high-energy electrons elicit optical Cherenkov photons within the patient’s body (Jarvis *et al* 2014, Zhu *et al* 2021). Practical applications of this modality have previously been evaluated and include entrance and exit dose verification (Zhang *et al* 2017), monitoring to detect treatment deviation (Alexander *et al* 2023), and retrospective analysis and comparison to the treatment plan. In proton therapy (PT), however, the Cherenkov output fluence alone was predicted to be too low for meaningful clinical use (Glaser *et al* 2014). Common therapeutic proton beam energies (70–250 MeV) are significantly lower than the Cherenkov emission production threshold (∼485 MeV in water, *n* = 1.33), and thus only the relatively few high-energy secondary electrons may contribute to the Cherenkov light generation (Helo *et al* 2014). Any light emission under therapeutic energies, to which Cherenkov generation is likely to contribute, has therefore been considered to be too low for meaningful evaluation. Recent advances in sensitive optical imaging technologies enable the detection of very low light levels but this remains to be validated for proton RT. This work aims to demonstrate optical imaging in conventional and UHDR proton therapy where it would provide similar visual real-time monitoring of beam delivery with respect to patient anatomy (Jarvis *et al* 2021) as that seen in photon or electron therapy.

Ultra-high dose rate (UHDR) irradiation (>40 Gy s^−1^) is being explored in order to elicit higher normal sparing while maintaining efficacy with respect to tumor control (Favaudon *et al* 2014). This increased therapeutic ratio at UHDR is known as the FLASH effect and is being used in human trials with PT^6^ 
^6^
FLASH Proton Therapy Clinical Trial Begins on Cancers Involving Bones in the Chest | Cincinnati Children’s. http://.cincinnatichildrens.org/news/release/2023/flash-clinical-trial.. Due to much shorter dwell times in UHDR PT, clinical surface guidance modalities and beam gating systems are not capable of monitoring or reacting to beam deviations at UHDR timescales. Therefore, FLASH PT could benefit from real-time optical imaging of relative entrance dose with respect to patient’s surface anatomy, for example to verify that shifts have not occurred immediately before or during beam delivery.

Previous work investigated proton irradiation in water tanks and discovered the presence of optical emission from water (Yamamoto *et al* 2015). This work used a long exposure cooled CCD camera setup which showed appreciable light emission under varying beam parameters (Yamamoto 2021, Yogo *et al* 2023). While such imaging shows promise for quality assurance (QA) applications, the low imaging rate and specific setup requirements are not suited to *in vivo* PBS proton treatment monitoring. This work demonstrates, for the first time, the detection and characterization of optical emission incited in *ex vivo* tissue by pencil beam scanning (PBS) PT beams under both conventional and UHDR conditions. By using a fast intensified CMOS (iCMOS) camera, the faint optical signal was amplified and recorded with adequate spatial and temporal resolution to allow for single spot monitoring. Further study was done to validate the ability of this imaging approach during proton UHDR RT. Finally, a tissue-mimicking plastic phantom was used to evaluate the signal strength dependence on proton beam energy.

## 2. Methods

### 2.1. Camera setup

**Figure 1.**
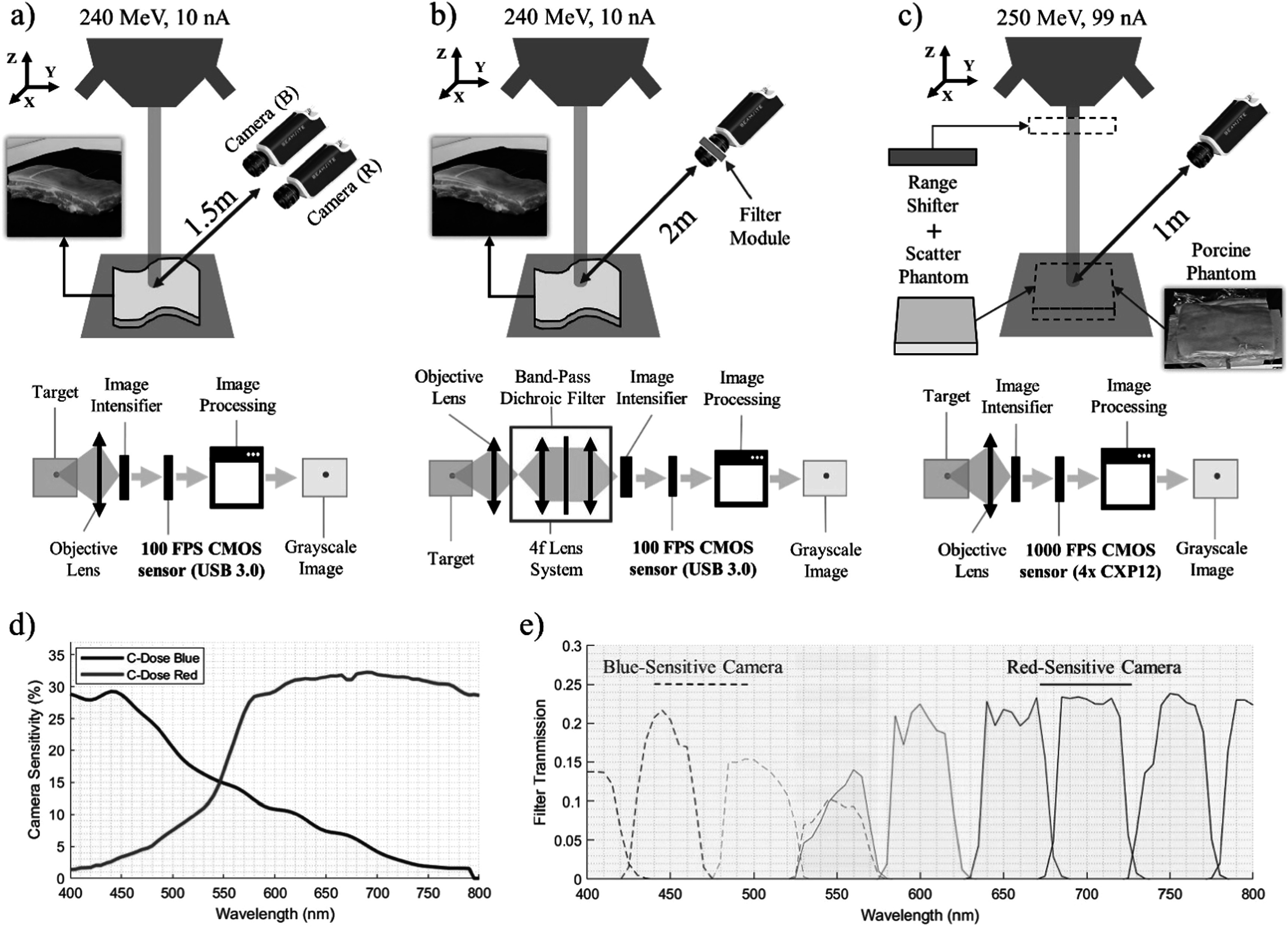
Experimental setups used to perform (a) broadband imaging of conventional PT from porcine tissue, (b) spectrally resolved imaging from porcine tissue, and (c) broadband imaging of UHDR PT from both tissue-mimicking optical phantom and porcine tissue to elucidate proton energy dependence and perform UHDR beam tracking, respectively. In all setups, the images are detected and amplified by image intensifier, the output from which is coupled to the CMOS sensor via a relay lens. Spectral imaging setup in (b) also uses blue and red sensitive detectors as in (a) and involves a 4 f imaging arrangement with an interchangeable dichroic band-pass filter located in common focal plane. (d) Measured spectral sensitivity curves for the cameras are shown along with (e) filter transmission values for spectrally resolved imaging.

As shown in figure 1, three different camera configurations were employed, allowing (a) conventional imaging (b) spectrally-resolved imaging, and (c) UHDR beam imaging and proton energy dependency study, using porcine tissue and tissue-mimicking plastic phantoms. Conventional and UHDR irradiations both used porcine phantoms from the same anatomical origin (ventral region of a mature pig) but collected at different times from different sources. The order of tissue components and relative composition was assumed to be the same based on qualitative observation. The conventional proton beam irradiations were imaged using an intensified CMOS (iCMOS) single channel camera (DoseOptics LLC) with USB 3.0 interface. The camera was equipped with a 50 mm, *f*/0.95 lens and a 1920 × 1200 pixel global shutter sensor. The camera recorded the pencil proton beam at a frame rate of 100 frames per second, which allowed for visualization of each individual spot. In UHDR study, we utilized a high-speed intensified CMOS camera (DoseOptics LLC) interfaced via 4× CXP-12 link to a PCIe frame grabber, enabling the image acquisition at 1000 fps (998 *μ*s frame duration and 2 *μ*s dead time) to resolve individual beam spots throughout the PBS plan delivery.

To resolve the emission spectrum of light emitted from tissue, we inserted an image relay system in a 4 f (4-focal length optical system) configuration between the main lens and an intensifier. The image relay system included a set of nine 1’ optical bandpass filters (400–800 nm central wavelength, 50 nm bandwidth, FGK01, Thorlabs) mounted in a manual filter exchanger. The wavelength-resolved images were recorded in sequential irradiations with the same beam profile. In order to optimize detection efficiency in the blue and red spectral range, blue-sensitive bi-alkali and red-sensitive GaAs intensifiers were employed, respectively. The 550 nm filter was used with both intensifiers and served as a reference point, allowing merging the spectra captured with the blue and red sensitive detectors. For all respective combinations of filters and intensifier type, we recorded the camera’s sensitivity utilizing a tunable, calibrated light source (TLS-6, Optometrics), allowing spectral sensitivity correction during image post-processing.

### 2.2. Proton irradiation procedure

#### 2.2.1. Conventional irradiation imaging

The RayStation treatment planning system was utilized to predict proton dose profile. A porcine tissue target consisting of skin, fat, and muscle tissue layers (approximate later thickness of 0.5, 10, 20 mm, resp.) was irradiated with a 240 MeV, 10 nA, 10 × 10 cm^2^ field PBS proton beam with single spot profiles measuring 5 mm FWHM at 1 standard deviation based on a gaussian curve.

#### 2.2.2. UHDR irradiation imaging

Porcine tissue was irradiated using a 250 MeV, 99 nA proton pencil beam over a 5 × 5 cm field size, administered via a Varian ProBeam system. The tissue layer composition consisted of approximately 0.5 mm of skin, 10 mm of fat, and 20 mm of muscle. Optical emission was imaged using an iCMOS single channel camera operating with a blue-sensitive intensifier at 1000 frames-per-second (fps).

To elucidate the impact of proton beam energy on the detected light intensity (figure 1(c)), we utilized a 2 cm thick slab of an optical tissue-mimicking plastic (Biomimic Optical Phantom, INO, Canada) as a target. The use of optically homogeneous phantom minimized the error due to unknown depth-weighing of the detected signal, which could have been present in the porcine phantom due to skin-fat-muscle layers. The nominal 250 MeV beam operating at a nozzle current of 99 nA was used to deliver a diamond-patterned field with a width and height of 5 cm across central axis. The planned PBS dose rate for this field was 54 Gy s^−1^ at isocenter (Folkerts *et al* 2020). Energy degradation was accomplished using 0 cm and 5 cm range shifters to achieve beam energies of 250 MeV and 229.1 MeV respectively. Beam energy values post range shifter filtering were obtained using the range–energy relationship outlined by Bortfeld and Schlegel (Bortfeld and Schlegel 1996). Optical emission was recorded using a spectrally unfiltered blue-sensitive iCMOS camera at 1000 fps. Radiochromic film dose measurements (EBT XD, Ashland) were done for each range shifter setup, further used for dose normalization during image processing.

### 2.3. Image processing

**Figure 2.**
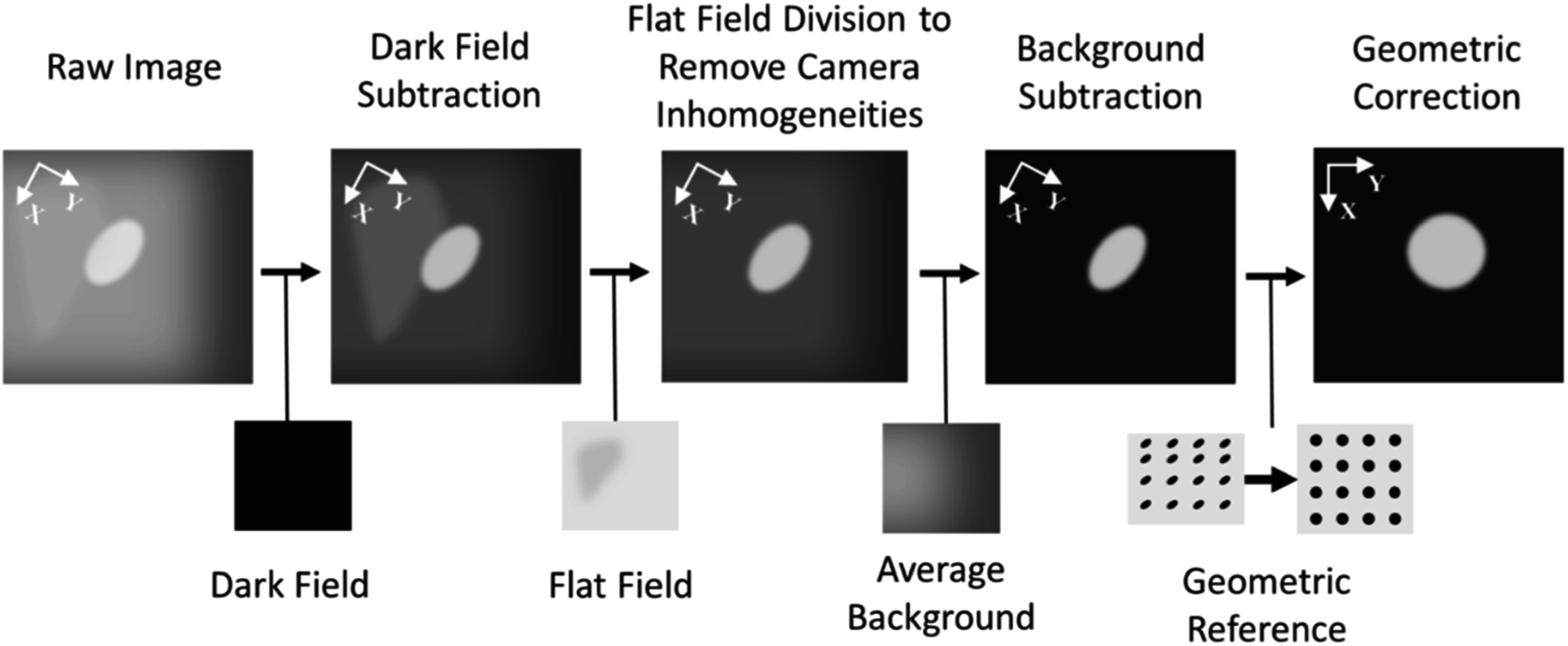
Image processing workflow. Raw images were subject to dark field subtraction, flat field normalization, background subtraction using the remaining signal frames from the initial acquisition, and geometric correction from pre-defined geometric reference.

Images were obtained and saved as 8 bit raw files directly upon acquisition. The range of frames bounded by the first and last instance of signal above the noise floor was isolated. The remainder of data was collected and used as a background image set. Pre-processing is outlined in figure 2 and was kept the same for all image data in conventional and UHDR imaging. For each signal frame, a dark field acquisition was subtracted, and a flat field correction was performed to remove any inhomogeneities in the intensifier and CMOS sensor light response. The background frames were averaged and subtracted from the image signal frames to isolate the light signal. A geometric reference consisting of a matrix of circles was imaged using the same experimental setup. The deformation in the reference frame was corrected using known internal positions and the same deformation matrix was applied to the signal data.

#### 2.3.1. Conventional spectral image processing

Optical emission images from the porcine target under single spot conventional PBS proton beam irradiation were collected and processed as outlined in figure 2. The emitted optical spectra were recorded and corrected using known filter quantum efficiencies to allow for direct comparison between spectral bands. Single spot profiles were isolated and fit to 2D Gaussian profiles; the central profile amplitudes were used as optical signal strength quantities for each wavelength measurement. Individual spot profiles were further normalized and compared with the expected Gaussian profile from the treatment planning system to evaluate the spatial profile agreement.

#### 2.3.2. UHDR scatter phantom image processing

Images obtained during UHDR PBS proton irradiation of the tissue-mimicking phantom were processed to identify the spot centroid locations. A surrounding region of 100 pixels (28 mm) around the center of each spot was isolated and the resulting cropped images were averaged. The average spot profiles were then fitted to a 2D double Gaussian function with a constant offset. For each energy, the spot intensity was integrated based on the fit function and compared in order to identify trends in energy dependence. During UHDR proton beam imaging, it was observed that the LUX19HS sensor’s light response showed a digitization error leading to value rounding in the 3.6–4.0 digital number (DN) range after background subtraction. This resulted in step-like features with constant intensity output (i.e. DN 3.8) spanning the entirety of the 3.6–4.0 DN range. As a result, pixels reporting the affected intensity levels were excluded from the fitting algorithm to maintain accuracy. Uncertainty bounds were derived using a numerical bootstrapping approach (1000 iterations) where the population of single spot frames considered for averaging was uniformly sampled with replacement. The mean values from the resulting distributions of integrated intensities were plotted with corresponding single standard deviation bounds in figure 5.

#### 2.3.3. UHDR porcine tissue image processing

Images obtained from UHDR PBS proton irradiation of the porcine tissue were saved in an 8 bit raw format and signal frames as well as background frames were isolated from the acquired stack. Both image sets underwent dark field subtraction and 2 × 2 spatial binning. Additional temporal and spatial median filtering were applied to the image data, using 2-frame and 2 × 2 pixel moving windows, respectively, in order to minimize spatio-temporal noise primarily caused by single photon response noise of the intensifier. An average background frame was subtracted from the signal images and single spot signal was isolated based on a global image threshold. Cumulative intensity map was calculated as a sum of all thresholded signal frames. Furthermore, the proton pencil beam spot map was created by localizing the centroid of the single spot signal for each signal frame and overlaying the coordinates with the cumulative image. Spots from consecutive frames that were separated by more than a Euclidean distance of 0.5 pixels were identified and marked as moving frames. Consecutive frames with Euclidean centroid separation of less than 0.5 pixels were identified and marked to represent a dwell position.

## 3. Results

### 3.1. Conventional PBS proton beam imaging and spot tracking

**Figure 3.**
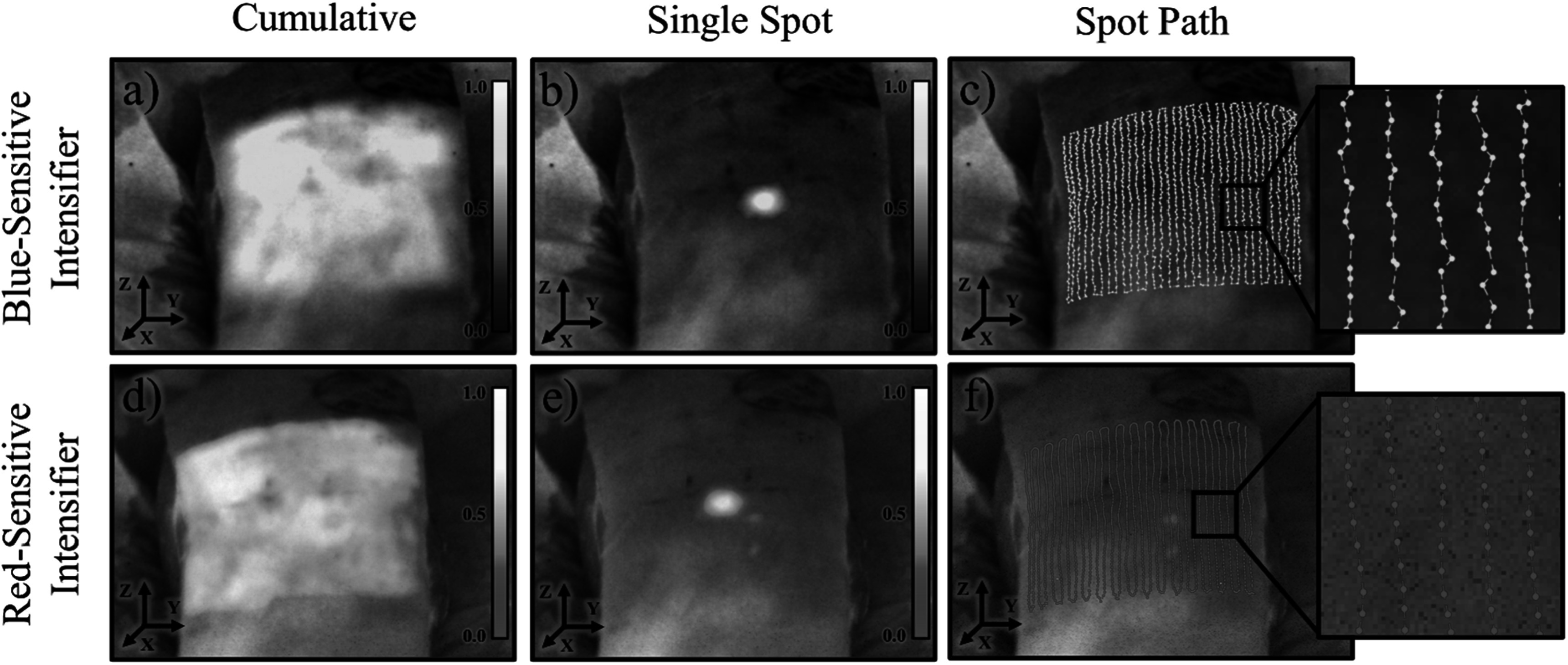
Spatio-temporally filtered images from the blue-sensitive intensified camera (top) and red-sensitive intensified camera (bottom) are shown over a grayscale background image of a porcine tissue target. Images taken from both cameras show normalized cumulative intensity maps (a), (d) followed by a normalized representative single-spot frame (b), (e). In (c) and (f) the spot paths are also shown with a magnified inset depicting a map of spot centroid positions, connected according to the sequence at which the spots were delivered and imaged.

The images of optical photon emission from an *ex vivo* porcine tissue irradiated by conventional proton beams are shown in figure 3, comparing the image quality of red-sensitive Gen3 and Blue-sensitive Gen2 based iCMOS cameras. The feasibility of a spatiotemporal mapping of single spots during a conventional proton field delivery (240 MeV, 10 nA) was demonstrated with frame-rate of 100 fps, as shown with an isolated single frame using both intensifiers. This is further supported by showing the temporal-resolved centroid identification in the spot path presented for both cases. The cumulative image shown indicates emission intensity variation which is spatially consistent in acquisitions by the two intensified cameras. Therefore, this variation is likely due to localized variation in surface pigment and differences in sub-surface tissue composition (Wickramasinghe *et al* 2023). With the presented setup, the red-sensitive intensified camera demonstrated an improved SNR = 4.85 as compared to blue-sensitive camera SNR = 2.42 due to the expected higher signal intensity at longer wavelengths.

### 3.2. Spectral analysis of PBS optical emission

**Figure 4.**
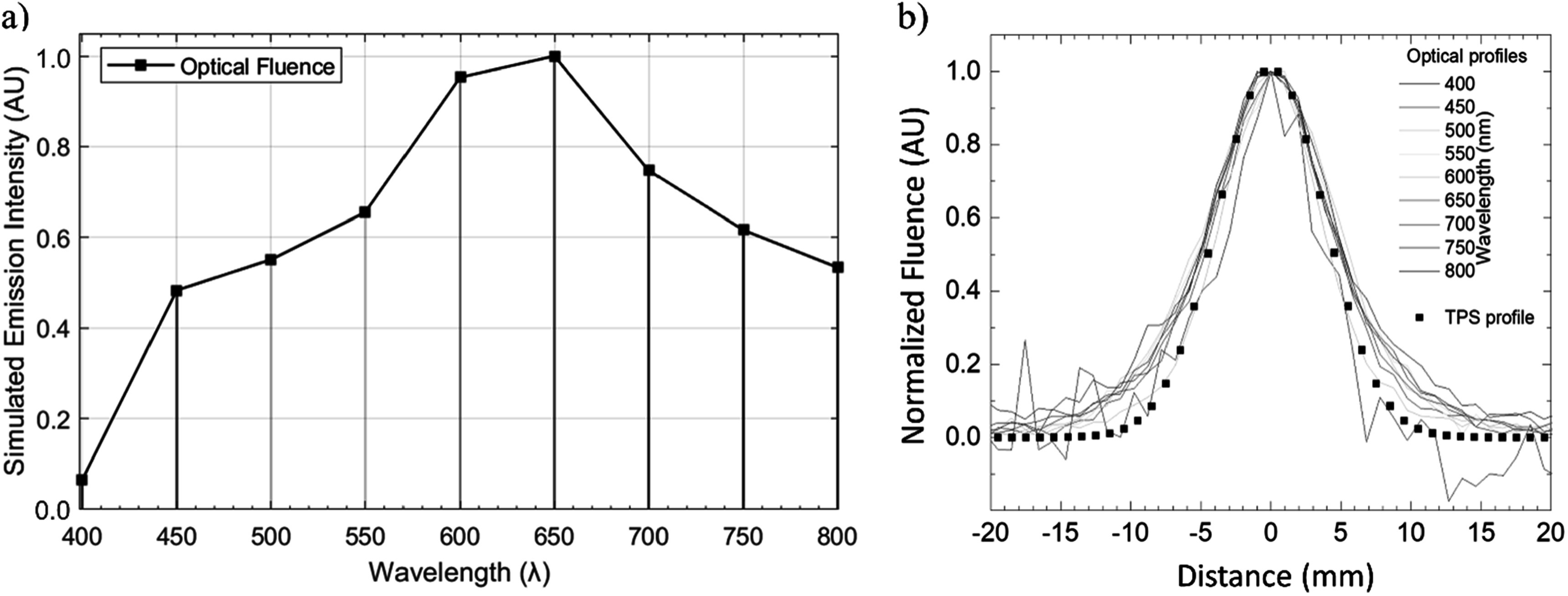
Imaged surface emission spectra across wavelengths in spectral range of 400–800 nm are plotted in 50 nm increments in (a). Normalized, temporally averaged spot profiles for each wavelength are compared to the treatment planning system (TPS) profile which indicates agreement of full width at half maximum (FWHM) within >90% for all spectral bands (b).

To evaluate the characteristic emission spectrum from skin, we performed spectrally resolved imaging of a single spot (250 MeV) repeatedly delivered to the same area of porcine tissue sample. The resulting emission spectrum, calibrated to absolute detector sensitivity, is plotted in figure 4. A broadband emission with maximum intensity at 650 nm and decreasing signal at lower wavelengths was observed, which is consistent with expected attenuation patterns of high fat and muscle tissue (Wickramasinghe *et al* 2023). We further compare the recorded spatial cross-sectional spot profiles for each spectral acquisition with the surface dose profile calculated with TPS. The FWHM of all spots agree within >90% accuracy of the TPS dose profile. This indicates that 100 fps iCMOS imaging of surface emission from PBS proton beams allows for adequate spatial single spot surface dose profiling. As expected, we observed an increased profile base at longer wavelengths likely due to optical scattering in the superficial fat tissue.

### 3.3. Influence of UHDR beam energy on relative light yield

**Figure 5.**
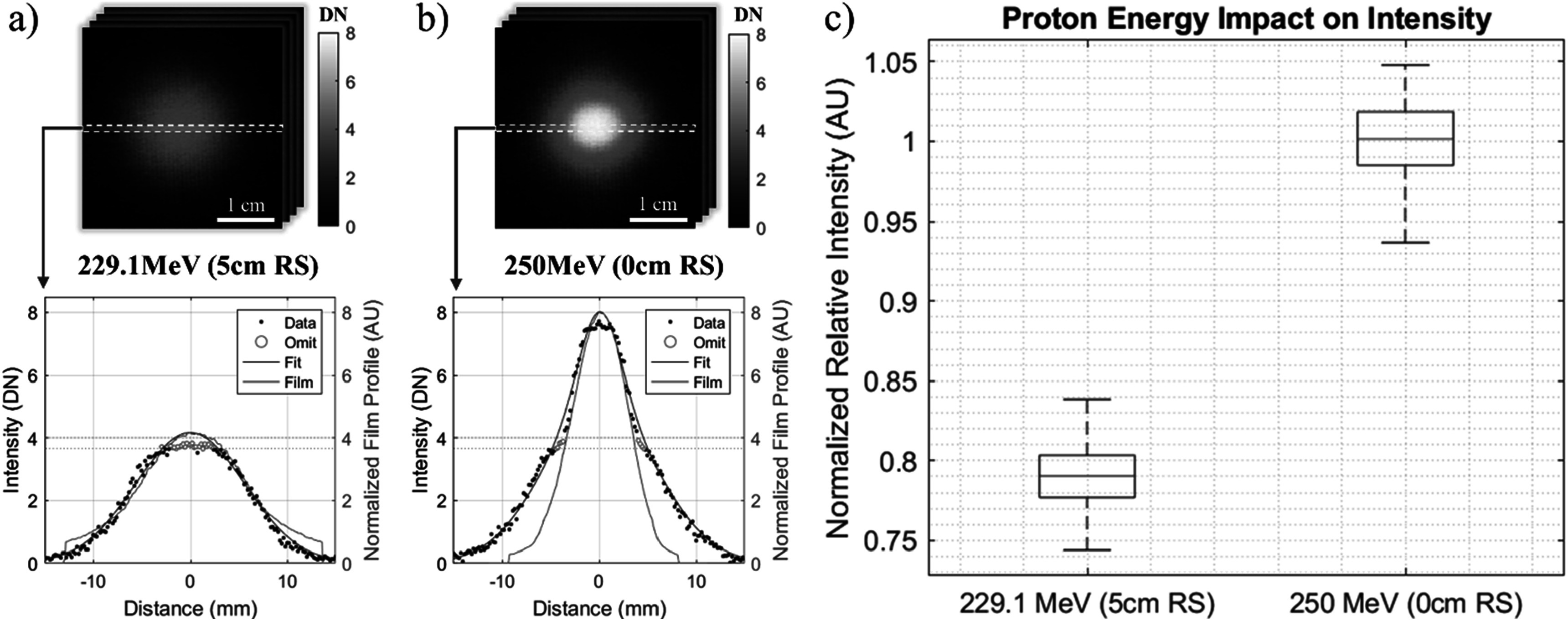
Difference of cross-sectional profiles of the average spot images for (a) 229.1 MeV and (b) 250 MeV demonstrate the dependence of surface emission signal intensity on proton beam energy. Intensity values affected by sensor loss of signal differentiability are marked red and were excluded from the fitting algorithm. (c) Dose normalized relative spot intensities are plotted with respect to the corresponding energies indicating the energy dependence of approx. 1%/MeV in the measured energy region.

Figure 5 shows the variation of optical output intensity on incident proton beam energy using the pristine 250 MeV beam and a 5 cm range shifter to degrade a nominal energy to 229.1 MeV. The average spot images are shown alongside the corresponding central axis cross-sections in figure 5(a) for each of the beam energies. Cross sectional plots include a single central slice of the averaged image as well as the central component of the corresponding double Gaussian fit. Film data was collected for each of the beam energies using a single spot delivery and the corresponding cross-sectional profiles were normalized and plotted alongside the image data. Good agreement between film and image data is seen at 229.1 MeV (*R* ^2^ = 0.979). 250 MeV comparison shows greater deviation (*R* ^2^ = 0.788) which may likely be attributed to the presence of higher spatial frequency components in the beam fluence distribution, and its light response being blurred due to optical scattering in the phantom. Figure 5 shows the integrated spot intensities for the respective energy profiles which were dose corrected, normalized, and sampled through 1000 iterations of a bootstrapping algorithm, varying the frames used for signal averaging to obtain uncertainty bounds for the integrated signal. Significant separation between average integrated intensities is observed, suggesting a strong energy dependence of the emitted signal intensity.

### 3.4. UHDR PBS proton beam spot imaging analysis

**Figure 6.**
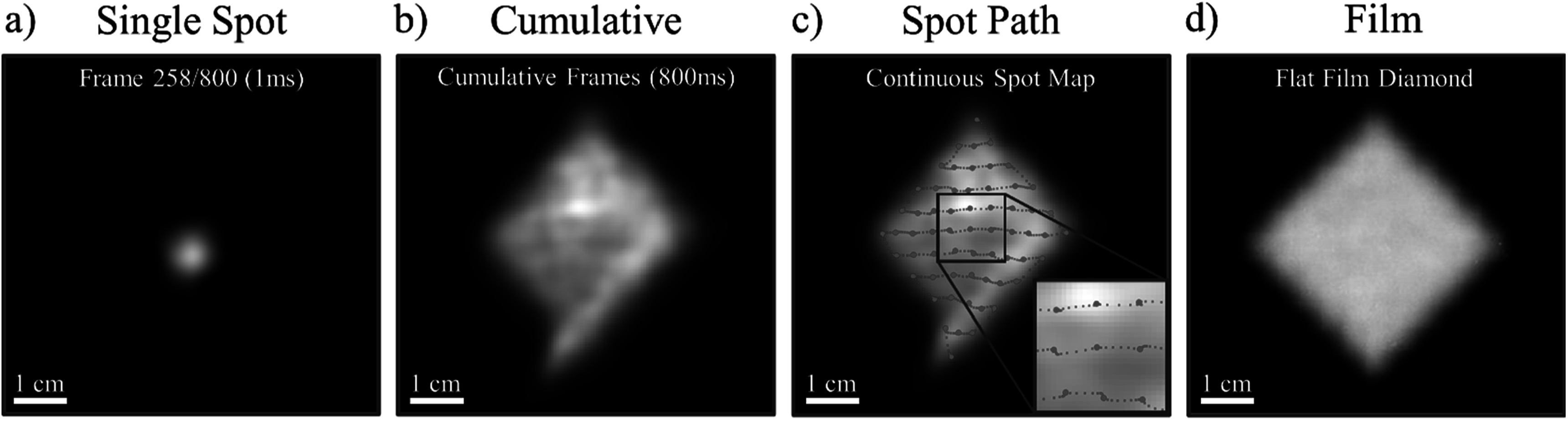
Demonstration of adequate temporal resolution for a single-spot imaging of optical emission during PBS UHDR therapy in tissue. A spot from an arbitrary frame 258 was isolated to indicate single-spot identification capability. Images are shown without localized geometric correction, thus the emission profile is affected by the tissue surface curvature. The cumulative profile from the full 800 ms acquisition in shown in (b) and is used as a background to show individual spot centroid localization in (c). The image inset (2× magnification) highlights the beam dwell spot locations (large red spots) along with centroids from beam during transition (small red spots). The total number of identified dwell point centroids matches the treatment plan in number per row (1, 3, 5, 7, 9, 11, 9, 7, 5, 3, 1) and in total (61). The same 5 × 5 diamond delivery to a film laid on flat solid water surface is shown (d) for reference.

Figure 6 shows optical emission from porcine tissue irradiated with a UHDR PBS proton beam (5 × 5 cm field, 250 MeV, 99 nA, 54 Gy s^−1^ at isocenter) imaged at 1000 fps. Geometric correction for local tissue curvature was not available which resulted in visible spatial deformation of the collected signal. A representative frame is isolated in figure 6(a), demonstrating single-spot delivery at 998 *μ*s frame duration, as recorded by the fast camera. Single spot raw signal average SNR was found to be 0.716 for full resolution images, and 3.003 after 2 × 2 binning. Figure 6(b) shows the cumulative emission from frames 1–800 (full delivery) with local intensity variations caused by lack of geometric correction, surface pigmentation, and local differences in tissue heterogeneity. Figure 6(c) further demonstrates the per-frame spot centroid localization of the full UHDR delivery. Areas with determined spot buildup (2+ frames with spot centroid location within 0.5 pixel Euclidean distance) were marked with large circles to indicate spot dwell positions. The total number of delivery positions matched the plan per row (1, 3, 5, 7, 9, 11, 9, 7, 5, 3, 1) and in total (61). A 5 × 5 diamond plan delivery onto flat film is shown as a reference for image distortion in figure 6(d).

## 4. Discussion

While previous studies have characterized the feasibility of intensified imaging of Cherenkov signal to monitor incidents during electron and photon radiotherapy (Decker *et al* 2022), the work in this study presents the first demonstration of tissue luminescence imaging during PBS proton treatment under conventional and UHDR irradiation. The nature of emission under proton irradiation is expected to differ from photon and electron irradiation. Compared to Cherenkov threshold for MV photon and MeV electron beams, the high mass of the proton significantly raises the threshold energy for direct Cherenkov light generation (∼485 MeV) which is significantly higher than what is commonly used in a clinically relevant range of proton beam energies (70–250 MeV). Secondary emission through energy transfer to neighboring electrons is, however, feasible and the maximum energy transfer can be approximated by classical momentum conservation as *E*_max_ = 4*E*_p_*(*m*_e_/*m*_p_), where *E*_p_ is the incident proton energy, *E*_max_ is the maximum energy of the scattered electron, and *m*_e_/*m*_p_ represents the electron and proton mass ratio. The maximum energy of secondary electrons is therefore found to be 545 keV for a 250 MeV primary proton beam, which is higher than the Cherenkov energy threshold for electrons in fat (195 keV, *n* = 1.45) and muscle (245 keV, *n* = 1.36). This suggests that generation of Cherenkov photons is still possible under these conditions, yet it is likely accompanied with other radiative processes as discussed below.

Imaging of porcine tissue during PBS proton irradiation at conventional and UHDR treatment dose rates showed detectable optical emission from regions of spot irradiation, in agreement with the treatment plan. The spot intensity was found to vary with wavelength across the whole range of 400–800 nm. The detected polychromatic emission spectrum was found to partially resemble the tissue-filtered spectrum from x-ray or electron induced Cherenkov emission (Alexander *et al* 2021), showing the expected peak around 650 nm and further agreement at higher wavelength. However, the lower wavelength disagreement between literature Cherenkov spectra (Alexander *et al* 2021) and experimental spectral data shown in figure 4(a) may suggest different phantom composition or additional sources of optical emission. Due to the low stopping power at the proton beam entrance, and fundamentally different particle-matter interaction, the population of secondary electrons with energies above Cherenkov threshold is significantly lower than in the case of photon and electron beams. Yamamoto *et al* (2021) demonstrated a presence of light emitted from water with low energy proton beams, suggesting that alternative mechanism(s) of luminescence, such as water fluorescence and/or autofluorescence likely add to the overall light yield. Water luminescence has been proposed in prior work with cooled CCD imaging of optical emission during water irradiation below the Cherenkov energy threshold, though at lower relative intensity (Hirano and Yamamoto 2019). Autofluorescence of light-emitting tissue constituents (lipofuscins, porphyrins) (Croce and Bottiroli 2014) is speculated to be yet another contributor to the observed light emission. Lastly, low energy Bremsstrahlung emission may also contribute to optical yield, despite having a low expected presence after tissue attenuation. Identification of the individual sources and physical mechanisms contributing to the observed light emission is warranted.

Inhomogeneity in optical signal during porcine tissue irradiation was also observed and is depicted in the non-uniform cumulative images in figure 3 most likely due to surface pigmentation as well as sub-surface variation in tissue composition (Hachadorian *et al* 2022, 2018). Corrections based on prior knowledge of optical transport in tissue may be implemented in future work to mitigate local intensity variation. Furthermore, surface geometric correction may be applied in future work to correct for variation is signal output due to local differences in surface angle to the beam or the camera.

A strong dependence of detected light on primary proton beam energy with the light loss of approximately 1%/MeV at a range of 229–250 MeV was observed. Previous Monte Carlo simulation studies (Glaser *et al* 2014) predicted a similar direct dependence and observed an increase in emission with energy suggesting that Cherenkov emission may contribute to the total signal. This increase is expected due to the linear relationship between proton energy and maximum energy transferred to secondary electrons. Future work may evaluate a finer energy distribution and pursue a quantitative comparison with simulation data following the same experimental setup. Furthermore, the local signal differentiation loss of the high speed sensor’s light response led to step-like features in the spot profiles which may be addressed in future work by optimizing sensor performance, using greater intensifier gain, or imaging with longer exposure times to gather more signal.

Optical emission from porcine tissue undergoing UHDR PBS proton beam irradiation was observed despite expected decrease in optical yield due to significant temporal constraints. Imaging at 1 kHz resulted in clear identification of all dwell points which matched the treatment plan. An average of 5 frames was also captured between individual dwell positions, showing capability for accurate monitoring of beam in transit. Although single spot identification was shown, the increase in frame rate resulted in lower optical yield per frame which led to decrease in SNR per spot. An optimal frame-rate may be found in future investigation to improve single-spot intensity while preserving appropriate temporal resolution for clinically relevant treatment and potential incidents.

Lastly, the imaging of optical emission was done under 240–250 MeV irradiation which is the upper limit of the clinically applicable energy range which indicates that the optical imaging method presents strong energy dependence. Future iterations require a more sensitive setup to capture lower yield emission from energies as low as 70 MeV.

## 5. Conclusion

Our study demonstrates the first imaging of optical emission in visible to near infrared wavelength range from tissue incited by scanned proton pencil beams. Our findings show that single-spot emission can be observed and imaged at both conventional and UHDR conditions with sufficient temporal resolution for continuous treatment monitoring. This development builds upon the already established *in vivo* Cherenkov imaging during conventional electron and photon EBRT and enables real-time imaging and spatiotemporal localization of beam entrance with direct reference to patient anatomy.

## Data Availability

Size of data set prevents it from being easily available, but it can be shared upon request. The data that support the findings of this study are available upon reasonable request from the authors.
